# Analysis of rare variants of autosomal‐dominant genes in a Chinese population with sporadic Parkinson’s disease

**DOI:** 10.1002/mgg3.1449

**Published:** 2020-08-14

**Authors:** Ran Zheng, Chong‐Yao Jin, Ying Chen, Yang Ruan, Ting Gao, Zhi‐Hao Lin, Jia‐Xian Dong, Ya‐Ping Yan, Jun Tian, Jia‐Li Pu, Bao‐Rong Zhang

**Affiliations:** ^1^ Department of Neurology College of Medicine Second Affiliated Hospital Zhejiang University Hangzhou Zhejiang China

**Keywords:** autosomal‐dominant genes, gene‐based analysis, Parkinson's disease, rare variants, sporadic

## Abstract

**Background:**

To date, several studies have suggested that genes involved in monogenic forms of Parkinson's disease (PD) contribute to unrelated sporadic cases, but there is limited evidence in the Chinese population.

**Methods:**

We performed a systematic analysis of 12 autosomal‐dominant PD (AD‐PD) genes (*SNCA*, *LRRK2*, *GIGYF2*, *VPS35*, *EIF4G1*, *DNAJC13*, *CHCHD2*, *HTRA2*, *NR4A2*, *RIC3*, *TMEM230*, and *UCHL1*) using panel sequencing and database filtration in a case‐control study of a cohort of 391 Chinese sporadic PD patients and unrelated controls. We evaluated the association between candidate variants and sporadic PD using gene‐based analysis.

**Results:**

Overall, 18 rare variants were discovered in 18.8% (36/191) of the index patients. In addition to previously reported pathogenic mutations (LRRK2 p.Arg1441His and p.Ala419Val), another four unknown variants were found in *LRRK2*, which also contribute to PD risk (*p* = 0.002; odds ratio (OR) = 7.83, 95% confidence intervals (CI) = 1.76–34.93). The cumulative frequency of undetermined rare variants was significantly higher in PD patients (14.1%) than in controls (3.5%) (*p* = 0.0002; OR=4.54, 95% CI = 1.93‐10.69).

**Conclusion:**

Our results confirm the strong impact of *LRRK2* on the risk of sporadic PD, and also provide considerable evidence of the existence of additional undetermined rare variants in AD‐PD genes that contribute to the genetic etiology of sporadic PD in a Chinese cohort.

## Graphical abstract

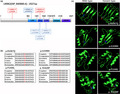

## 1 INTRODUCTION

Parkinson's disease (PD) is the most common movement disorder (Alves, Forsaa, Pedersen, Dreetz Gjerstad, & Larsen, 2008), with a global prevalence of 6.2 million cases in 2015 that is estimated to rise to 12.9 million by 2040 (Dorsey & Bloem, 2018). PD is defined by two major neuropathological hallmarks: loss of dopaminergic neurons in the substantia nigra pars compacta of the midbrain and the presence of Lewy bodies (LB) (Postuma et al., 2015). However, the etiology of PD has not been fully elucidated. In addition to increasing age and exposure to environmental risk factors, genetics also play an important role in the pathology of PD (Blauwendraat, Nalls, & Singleton, 2019).

Several genome‐wide association studies (GWASs) have revealed a considerable number of candidate variants associated with PD, although some were first discovered in family‐based linkage studies; however, many of these genes were successfully replicated in common sporadic PD patients (Simon‐Sanchez et al., 2009; Spataro et al., 2015), indicating that genes involved in monogenic forms of the disease also act as susceptibility factors in the unrelated sporadic form of PD. Despite these discoveries, much of the genetic contribution to PD remains unexplained (Spataro et al., 2015), in part, because when GWASs began, the field was dominated by the simple common disease–common variant hypothesis. GWAS effectively represent common genetic variants with a typical minor allele frequency (MAF) >1%–5% (Lee, Abecasis, Boehnke, & Lin, 2014). Analyses of rare variants (MAF <1%) may explain additional disease risk or trait variability (Gibson, 2012) especially in diseases with complex traits such as PD.

Several studies have indicated an association between rare variants of Mendelian genes and sporadic PD in different populations (Benitez et al., 2016; Lesage & Brice, 2012; Spataro et al., 2015; Tan et al., 2019); however, there is limited evidence in the Chinese population. Recently, Yang et al. (2019) identified several rare variants of seven autosomal‐dominant PD (AD‐PD) genes consisting of *SNCA* (OMIM No. 163890), *LRRK2* (OMIM No. 609007), *GIGYF2* (OMIM No. 612003), *VPS35* (OMIM No. 601501), *EIF4G1* (OMIM No. 600495), *DNAJC13* (OMIM No. 614334), and *CHCHD2* (OMIM No. 616244) in a case‐control study with Chinese ethnic background, indicating the possible contribution of other AD‐PD genes. In this study, we investigated these seven AD‐PD genes and five additional previously reported monogenic AD‐PD genes, including *HTRA2* (OMIM No. 606441), *NR4A2* (OMIM *No*. 601828), *RIC3* (OMIM No. 610509), *TMEM230* (OMIM No. 617019), and *UCHL1* (OMIM No. 191342), (Blauwendraat et al., 2019), to further illustrate the association between monogenic genes and sporadic PD patients in Chinese population.

## 2 METHODS

### 2.1 Ethical compliance

This study was conducted in accordance with the Declaration of Helsinki with formal approval obtained from the ethics review boards of the Second Affiliated Hospital of Zhejiang University. All participants provided written informed consent to genetic analysis and disclosure of medical information.

### 2.2 Subjects

In total, 191 sporadic PD cases (aged 16–82 years) and 200 ethnicity‐matched controls were recruited from the outpatient neurology clinics of the Second Affiliated Hospital of Zhejiang University (China) between January 2016 and June 2019. All subjects were examined by at least two neurology physicians specializing in movement disorders. Inclusion criterion referred to the diagnosis of PD based on the clinical criteria defined by the Movement Disorder Society (Postuma et al., 2015). Patients with secondary Parkinsonism and other forms of atypical Parkinsonism or with a family history of PD were excluded.

### 2.3 Sample preparation and sequencing

The quality and concentration of genomic DNA extracted from peripheral whole blood samples using standard procedures were assessed with a Qubit 3.0 Fluorometer (Life Invitrogen). Fragmented genomic DNA was captured by a customized array designed to target all exons, splicing sites, and flanking intronic sequences of 12 selected genes (as shown in Table S1). Sequencing was conducted as 150‐bp paired‐end runs on an Illumina NovaSeq 5000 system to an average depth of coverage >300‐fold. Sequence reads were mapped to the human assembly GRCh37/hg19 (GCA_000001405.1) using a Burrows–Wheeler–Aligner (BWA) (Li & Durbin, 2009), and variant calling was conducted using SAMtools (Li, 2011), followed by variant annotation using ANNOVAR (Yang & Wang, 2015).

### 2.4 Variant filtration

To identify candidate rare variants, we adopted a three‐level filtration algorithm. First, we required a MAF <1% or “not available” for variants in the Genome Aggregation Database (gnomAD), the 1000 Genomes Project (May 2019), and the Exome Aggregation Consortium (ExAC) (Genomes Project et al., 2015); otherwise, variants with unbalanced reads (variant allele <25%) and regions covered by <5× reads were eliminated. Second, we selected non‐synonymous substitutions including missense and nonsense mutations as well as small insertions and deletions, which are considered to be the most likely to cause loss‐of‐function of the encoded protein (Adzhubei et al., 2010; Fu et al., 2013). Third, for further prioritization, all selected non‐synonymous variants were analyzed with dbNSFP (X. Liu, Wu, Li, & Boerwinkle, 2016) (version 3.5) and CADD (Rentzsch, Witten, Cooper, Shendure, & Kircher, 2019). We retained only variants previously reported to be pathogenic in ClinVar (Landrum & Kattman, 2018) (www.ncbi.nlm.nih.gov/clinvar/) as well as rare variants that had CADD phred scores >15 or REVEL scores >0.5 or MetaL ranking score >0.75 or predicted to be damaging in the three most commonly used in silico pathogenicity prediction applications (SIFT, PolyPhen‐2, and mutationTaster) according to previous studies (Richards et al., 2015; Tian et al., 2018). All candidate variants were validated by Sanger sequencing, with primers designed using Primer3 (Koressaar et al., 2018). Carriers of candidate variants were screened using Multiplex Ligation‐Dependent Probe Amplification to exclude confounders from common gross deletions or duplications. The three‐dimensional (3D) protein structures of the wild‐type and variant‐type proteins were predicted using Phyre2 (Kelley, Mezulis, Yates, Wass, & Sternberg, 2015) and visualized by PyMOL (The PyMOL Molecular Graphics System, Version 2.3, Schrödinger, LLC).

### 2.5 Statistical analysis

Demographic characteristics were depicted as the means ± standard deviation (SD) and compared using Student's *t*‐tests. Sex‐related variables were assessed using Chi‐square tests. For all candidate rare variants, we calculated the proportion of carriers and assessed Hardy–Weinberg equilibrium with the Chi‐square test in both cases and controls. The subgroup of candidate variants categorized as unclear pathogenicity was evaluated with gene‐based burden analysis. Associations between rare variants and sporadic PD were analyzed using Chi‐square tests or Fisher's exact test, odd ratios (OR), and 95% confidence intervals (CI) (Li & Leal, 2008). All analyses were conducted using SPSS version 26 (IBM, Armonk, NY, USA). A two‐tailed *p*‐value of 0.05 was set as a nominal significance threshold.

## 3 RESULTS

### 3.1 Summary of demographic data

We screened for 12 AD‐PD genes consisting of three genes previously reported to contain mutations robustly associated with PD (*SNCA*, *LRRK2*, and *VPS35*) and nine genes associated with PD with low confidence (*GIGYF2*, *EIF4G1*, *DNAJC13*, *CHCHD2*, *HTRA2*, *NR4A2*, *RIC3*, *TMEM230*, and *UCHL1*) in 191 Chinese sporadic PD patients and 200 unrelated controls. Our cohort comprised 55% early onset PD (EOPD) with the age at onset of 43.72 ± 7.13 years and 45% late‐onset PD (LOPD) with the age at onset of 60.32 ± 7.96 years. Overall, the mean age of onset at enrollment was 51.15 ± 11.16 years for cases (55% males) and there was no significant difference compared with the mean age at onset of controls at 49.49 ± 9.86 years (53% males) (Table 1).

**Table 1:** Summary of demographic data

Series	N	AAO mean ± *SD* (range) in years	Male:Female ratio
Total PD	191	51.15 ± 11.16	106:85
EOPD (AAO ≤ 50)	106	43.72 ± 7.13	56:50
LOPD (AAO > 50)	85	60.32 ± 7.96	50:35
control	200	49.49 ± 9.86	106:94

Abbreviations: AAO, age at onset; EOPD, early onset Parkinson's disease; LOPD, late‐onset Parkinson's disease; N, number.

### 3.2 Overview of candidate rare variants

**Figure 1:**
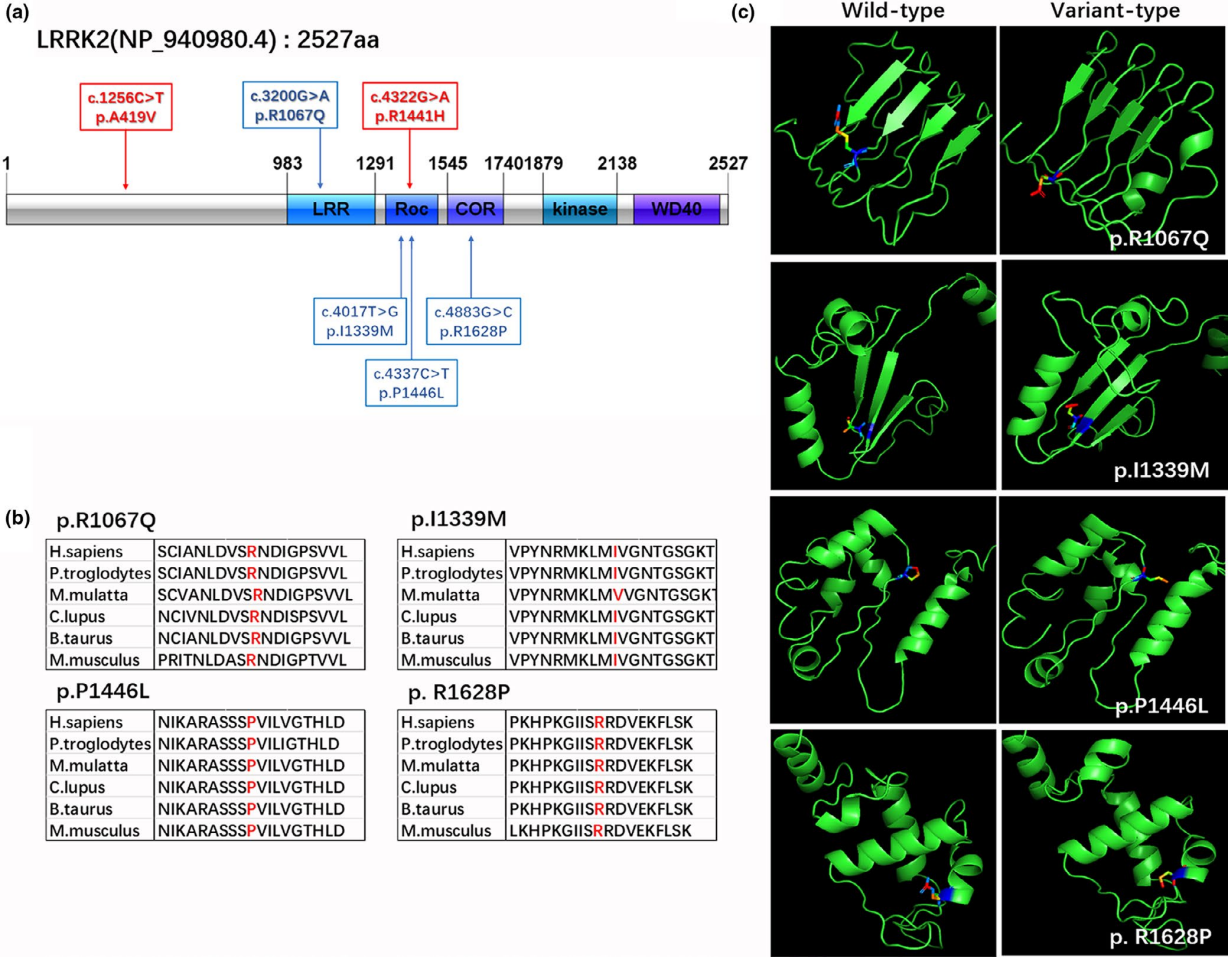
Schematic representation of *LRRK2* and its variations. (a) *LRRK2* contains 51 exons encoding a 2527‐amino acid protein with a leucine‐rich repeat domain, a Ras/GTPase (ROC) domain, and a protein kinase domain. The previously identified pathogenic variations in our cohort are indicated by red arrows and the other candidate variations are indicated by blue arrows. (b) The position and surroundings of unclear variants were highly conserved across different species. (c) The 3D structures of wild‐type and variant‐type proteins. Protein models are shown as secondary structures. Variant sites are shown as amino acid sequences

We validated candidate variants after the three‐level filtration in this cohort. A total of 18 rare non‐synonymous coding variants were validated in 18.8% (36/191) of sporadic patients. Among them, 33.3% (6/18) of the variants were found in *LRRK2* with the locations presented in Figure 1, and 55.6% (10/18) in *CHCHD2*, *DNAJC13*, *GIGYF2*, *NR4A2*, and *SNCA*, all of which had two candidate variants. The rest of these variants were singletons, located in *HTRA2* and *EIF4G1*. Despite high sequencing coverage, no rare variants were found in *RIC3*, *TMEM230*, *UCHL1*, and *VPS35* in our cohort (Table 2). Remarkably, none of the cases of candidate variant carriers were affected by common gross deletions and duplications.

**Table 2:** Summary of candidate variants after three‐level filtration in our cohort

Gene	Chromosome position^a^	dbsnp150	Amino Acid Change	Freq.GnomAD	Freq.1000G	Freq.ExAC	CADD‐phred	REVEL_score	MetaLR_rankscore	In silico tools			GERP++
										SIFT	PolyPhen‐2	MutationTaster	
CHCHD2	56172037	rs864309650	T61I	NA	NA	NA	28.1	0.73	0.72	D	D	D	5.62
	56174102	rs142444896	P2L	9.70E‐03	4.39E‐03	7.50E‐03	24.7	0.28	0.57	D	D	D	4.83
DNAJC13	132181345	novel	L583S	4.47E‐05	NA	7.42E‐05	28.3	0.47	0.66	D	D	D	5.96
	132222104	rs145101163	R1588H	8.12E‐06	NA	NA	26.6	0.31	0.47	D	D	D	5.5
EIF4G1	184044759	novel	R1139H	1.63E‐05	NA	3.31E‐05	34	0.2	0.75	D	D	D	5.72
GIGYF2	233684598	rs757005602	E811A	2.80E‐04	2.00E‐04	3.00E‐04	22.8	0.47	0.85	T	D	D	5.25
	233712060	rs200216092	P1155T	3.26E‐05	NA	3.30E‐05	24.4	0.82	0.84	D	D	D	5.54
HTRA2	74757881	rs200036604	T215M	1.22E‐05	NA	8.31E‐06	22.7	0.28	0.84	D	P	D	4.94
LRRK2	40646786	rs34594498	A419V	4.80E‐04	1.40E‐03	5.00E‐04	24.3	0.33	0.18	D	D	A	5.12
	40704252	rs74681492	P1446L	3.24E‐05	NA	NA	34	0.83	0.84	D	D	D	5.63
	40704237	rs34995376	R1441H	1.99E‐04	9.98E‐04	2.00E‐04	28.8	0.64	0.82	D	D	A	5.64
	40702326	rs773070538	I1339M	1.76E‐03	6.39E‐03	1.70E‐03	24.8	0.59	0.76	D	D	D	5.46
	40713845	rs33949390	R1628P	4.06E‐06	NA	8.24E‐06	27.8	0.55	0.69	D	D	D	5.54
	40692148	rs111341148	R1067Q	9.76E‐05	5.99E‐04	1.00E‐04	34	0.28	0.49	D	D	D	5.85
NR4A2	157182438	rs201003462	V539M	8.14E‐06	NA	NA	24.8	0.63	0.97	T	D	D	6.06
	157182309	rs753783927	V582M	1.63E‐05	NA	1.69E‐05	26.8	0.4	0.76	D	D	D	6.07
SNCA	90756775	novel	V15D	8.14E‐05	2.00E‐04	9.90E‐05	30	0.93	0.91	D	D	D	4.28
	90650354	rs191055637	M127I	NA	NA	NA	25.4	0.49	0.85	T	P	D	4.31

Note:

ExAC, Exome Aggregation Consortium; GnomAD, Genome Aggregation Database; PolyPhen‐2, SIFT, and MutationTaster: The in silico tools more commonly used for missense variant interpretation.

A, disease‐causing automatic; D, damaging or disease‐causing; NA, not available; P, possibly damaging; T, tolerated. GERP++ is a score for the conservation of the amino acid: scores >3 can be considered as highly conserved.

^a^ Position on Genome Reference Consortium human genome build 37 (GenBank assembly accession: GCA_000001405.1).

### 3.3 Rare non‐synonymous variants

Among the candidate variants, 16.7% (3/18) were previously reported and known as pathogenic in the ClinVar databases; these variants consisted of *LRRK2* p.Arg1441His, *LRRK2* p.Ala419Val, and *CHCHD2* p.Thr61Ile. In our cohort, *LRRK2* p.Ala419Val was the most common pathogenic mutation, accounting for 4.2% (8/191) of cases. Regarding *CHCHD2* p.Thr61Ile, the carrier of this mutation also carried the *LRRK2* p.Ala419Val mutation.

A total of 66.7% (12/18) of the candidate variants have been reported previously, although their pathogenicity is unknown; and 16.7% (3/18) were novel with unknown significance, which were located in *DNAJC13* p.Leu583Ser, *SNCA* p.Val15Asp, and *EIF4G1* p.Arg1139His (Table 2). To eliminate interference from known mutations and investigate whether additional rare variants in specific genes contribute collectively to PD risk, we performed a gene‐based analysis of the unclear variants. The cumulative frequency of these variants was significantly higher in PD patients (14.1%) than that in controls (3.5%) (*p* = 0.0002; OR = 4.54, 95% CI = 1.93–10.69), suggesting that most of these variants are likely to be true risk factors for PD (Table 3).

**Table 3:** Gene‐based analysis for rare non‐synonymous variants

Gene	All rare variants involved					Remove known mutations				
	patients/191	controls/200	*p* value	OR (95%CI)	Power	patients/191	controls/200	*p* value	OR (95%CI)	Power
LRRK2	23	2	0.000*	13.55 (3.15‐58.33)	0.94	14	2	0.002*	7.83 (1.76‐34.93)	0.77
CHCHD2	2	1	0.54	—	—	1	1	0.97	—	—
DNAJC13	3	0	0.12	—	—	3	0	0.12	—	—
EIF4G1	1	2	0.59	—	—	1	2	0.59	—	—
GIGYF2	2	0	0.24	—	—	2	0	0.24	—	—
HTRA2	1	0	0.49	—	—	1	0	0.49	—	—
NR4A2	3	1	0.29	—	—	3	1	0.29	—	—
SNCA	2	1	0.54	—	—	2	1	0.54	—	—
All genes	36	7	0.0002*	6.40 (2.77‐14.79)	0.99	27	7	0.0002*	4.54 (1.93‐10.69)	0.93

Abbreviation: 95% CI, 95% confidence intervals.

* *p* < 0.05 was considered significant and marked in bold font.

Of the unclear variants, 26.7% (4/15) were located in *LRRK2* (p.Pro1446Leu, p.Ile1339Met, p. Arg1628Pro, and p.Arg1067Gln). The overall frequency of *LRRK2* undetermined variants was much higher in PD patients (7.3%) than in controls (1%) (*p* = 0.002; OR = 7.83, 95% CI = 1.76‐34.93) and remained statistically significant after the Bonferroni correction (*α* = 0.05/8 = 0.0063). Among these variants, *LRRK2* p.Arg1628Pro was the most common risk variant and was significantly enriched in cases (5.2%) when compared to controls (0.5%) (*p* = 0.005; OR = 10.99, 95% CI = 1.39–86.74, Table S2). Remarkably, two variants located in DNAJC13 (p.Leu583Ser and p.Arg1588His), two variants located in GIGYF2 (p.Glu811Ala and p.Pro1155Thr), and one variant located in HTRA2 (p.Thr215Met) were present in 3.1% (6/191) of the PD individuals but absent in the controls. However, some candidate variants including p.Pro2Leu of CHCHD2, p.Arg1139His of EIF4G1, p.Val582Met of NR4A2, and p.Val15Asp of SNCA were present on both cases and controls. It should be noted that there was a greater number of carriers of *EIF4G1* p.Arg1139His in the control group than in the PD patients (Table 3).

## 4 DISCUSSION

The phenotype of autosomal‐dominant inheritance familial PD, which is similar to sporadic PD, suggests that monogenic and sporadic forms of the disease are etiologically related (Simon‐Sanchez et al., 2009). In this study, we found 18 rare variants in 18.8% of sporadic PD patients. One third of these variants located in the *LRRK2* gene were involved in 12.0% cases, which showed the strong effect of *LRRK2* on the risk of sporadic PD. Additionally, there were significantly more carriers of candidate variants in PD cases than in controls, implicating these variants as true risk factors for PD.

*LRRK2* was first identified in an autosomal‐dominant inheritance in late‐onset Parkinsonian families in 2004 (Zimprich et al., 2004). Variants in different domains of *LRRK2* have been identified in both familial and sporadic PD in different ethnic populations (Berg et al., 2005; Di Fonzo et al., 2005; Gilks et al., 2005). In our study, six candidate variants of *LRRK2* were found to be located mainly in functional domains. In addition, 12.0% (23/191) of patients found to carry those variants exhibited typical Parkinsonian symptoms, with most showing initial motor features of slowly progressive asymmetric tremor at rest or bradykinesia (Table 2). As previously reported, the *LRRK2* p.Gly2019Ser mutation is thought to be the most frequent (Lunati, Lesage, & Brice, 2018); however, we did not find any carrier of this mutation in our cases. The most likely reason for this is that p.Gly2019Ser exists as a founder variant mainly in Eastern European Jews and North African Berbers, but not in Asian populations (Hulihan et al., 2008; Tan et al., 2010; Thaler, Ash, Gan‐Or, Orr‐Urtreger, & Giladi, 2009). We found only one case carrying the p.Arg1441His mutation, a pathogenic variant first identified in a Taiwanese PD family (Mata et al., 2005). *LRRK2* P.Arg1441His occurred adjacent to two previously reported pathogenic mutations, p.Arg1441Cys and p.Arg1441Gly, identified as a 4322G‐A transition in exon 31 of *LRRK2*. This mutation resulted in an Arg1441His substitution in the Ras/GTPase (ROC) domain, which may impair the regulation of kinase activity (Gilsbach & Kortholt, 2014). Since Mata et al. (2005) identified the first p.Arg1441His carrier with an Asian ethnic background among 100 affected probands with a family history of Parkinsonism, familial PD carriers of diverse ethnicity have been identified in follow‐up studies (Ferreira et al., 2007; Spanaki, Latsoudis, & Plaitakis, 2006; Zabetian et al., 2005). Subsequently, a large case‐control study also confirmed one Asian PD carrier of p.Arg1441His (Ross et al., 2011). To date, the association of *LRRK2* p.Arg1441His in sporadic PD is supported by limited data; thus, our study provides further evidence in support of this. Regarding *LRRK2* P.Ala419Val, the most common pathogenic variant in this cohort was classified as pathogenic in ClinVar. Ross et al. reported a significant difference in the prevalence of p.Ala419Val between PD patients and controls in an Asian population (Ross et al., 2011), and this was confirmed by Guo et al. (Li et al., 2015) in a Chinese population. Guo and colleagues also found that p.Ala419Val especially affected patients with EOPD, which is consistent with our data (Table 2).

The most common risk variant was also found in *LRRK2* p.Arg1628Pro, located in the COR domain. The substitution of a highly basic polar arginine (R) with a neutral nonpolar proline (P) is likely to cause a conformational change in the secondary structure of the LRRK2 protein. In a study of 1986 individuals from Taiwan and Singapore, Wu et al. (Ross et al., 2008) demonstrated that p.Arg1628Pro increased the risk for PD, although non‐Asian carriers were not identified in previous studies, indicating an important Asian genetic specificity. Later, Wu showed that this variant was also associated with PD in Chinese patients (Tan et al., 2010), although a subsequent study by Deng et al. (Yuan et al., 2016) failed to find statistically significant differences in either genotypic or allelic frequency of p.Arg1628Pro between patients and controls in a Chinese population; our results corroborated Wu's findings. The discrepancies in the association between genetic variants and the presence of PD in the same or different populations may explain, to a large extent, the inconsistency in the results of these studies. Overall, our study further confirmed the association between *LRRK2* and idiopathic PD in the Chinese population.

*SNCA*, which was the first recognized AD‐PD gene (Golbe, Di Iorio, Bonavita, Miller, & Duvoisin, 1990), encodes α‐synuclein, the primary component of LB (Goedert, 2001). Although we did not find any known pathogenic mutations in *SNCA* in this study, we identified one novel variant, p.Val15Asp, and one unclear variant, p.Met127Ile, located between two phosphorylation sites with high conservation across variable species. None of the controls carried p.Met127Ile, suggesting that this variant is associated with susceptibility to PD.

In *CHCHD2*, we discovered one unknown variant, p.Pro2Leu, and one pathogenic variant, p.Thr61Ile, previously reported by Funayama et al. (2015) in two unrelated Japanese families segregated with disease. Although almost all the subsequent studies in the Chinese population suggested that *CHCHD2* mutations are not a common cause of PD in Chinese familial or sporadic cases (Gao et al., 2017; Liu et al., 2015; Shi et al., 2016), our findings provide new evidence of the role of this gene in susceptibility to sporadic PD in China. Nevertheless, this association needs further confirmation in additional series.

In our study, we found a novel variant, *DNAJC13* p.Leu583Ser, carried by one patient and an unclear variant, *DNAJC13* p.Arg1588His, carried by two patients. Although these two variants are not located in the functional regions of *DNAJC13*, neither were carried by controls, suggesting these variants may contribute to the risk of disease by regulating gene function. Vilarino‐Guell et al.^9^, ^2014^) identified a heterozygous missense variant of *DNAJC13* not only in both familial and sporadic PD patients, but also asymptomatic carriers. In addition, Tan et al. (Foo et al., 2014) found that coding variants of *DNAJC13* were extremely rare and present in healthy controls without enrichment in PD cases in a Chinese population. Since the data in the Chinese population are limited, the contribution of *DNAJC13* variants to the risk of PD remains plausible. However, considering the rare frequency of *DNAJC13* variants and the inconsistency of previous reports, it can be speculated that the contribution of this gene to sporadic PD is very limited in the Chinese population.

Despite adopting a three‐level filtration of candidate variants designed to identify the most pathogenic sites, the novel variant EIF4G1 p.Arg1139His identified in one of the patients was also present in two of the control individuals; therefore, the importance of this variant should be considered with caution. This paradoxical result may be due to the limited sample size and the low frequency of *EIF4G1* in Asian populations (Zhao et al., 2013). It is also interesting to note that loss‐of‐function variants of known pathogenic AD‐PD genes, such as *LRRK2* and *SNCA*, were also identified in healthy controls (Hernandez, Reed, & Singleton, 2016). The most likely explanation for this phenomenon is that these variants cause disease when inherited in the form of compound heterozygotes or homozygotes or with risk variants of other genes, which will confound risk prediction.

We discovered two *NR4A2* variants (p.Val539Met and p.Val582Met) with unknown pathogenicity. *NR4A2* has been widely studied in the Chinese population. Xu et al. (2002) and Zheng, Heydari, and Simon (2003) reported associations between homozygosity or heterozygosity for a variant in the intron of this gene and PD. Many follow‐up studies (Le et al., 2003; Liu et al., 2013; Tan et al., 2004) revealed the presence of *NR4A2* variants in both familial and sporadic cases of PD among various populations. Our study provided more evidence for the association between rare variants of *NR4A2* and sporadic PD in Chinese population. Other selected genes, GIGYF2 and HTRA2, were found to have one or two unclear variants carried by only PD patients but not by controls. Since no rare variants were found in *RIC3*, *TMEM230*, *UCHL1*, and *VPS35* in our cohort, further investigations are required to clarify the role of those genes in susceptibility to PD in the Chinese population.

Some limitations of our study should be noted. Due to the moderate sample size, we detected only a small number of rare variants in these genes. Therefore, the value of individual single‐nucleotide polymorphisms (SNPs) in predicting risks is limited and combination with multiple low‐penetrance SNPs may increase the predictive power. Although we performed gene‐based analysis in candidate variants, some genes present in a limited number of carriers do not meet the requirements for statistical analysis and studies with larger samples are needed to clarify our findings.

## 5 CONCLUSIONS

In our study, 18 rare non‐synonymous coding variants were validated in 18.8% (36/191) of index patients. Among them, most of the variants were found in *LRRK2*, indicating the strong impact of *LRRK2* on sporadic PD risk in the Chinese population. Unclear rare variants in *DNAJC13*, *GIGYF2*, and *HTRA2* may also confer susceptibility to PD risk since none of the controls were affected. In summary, our findings validate the etiological relationship between rare variants of AD‐PD genes and sporadic PD cases in an eastern Chinese population. These results must be interpreted with caution considering our moderate sample size and insufficient power, and further studies in larger samples are required to clarify this relationship.

## CONFLICT OF INTEREST

The authors disclose no conflict of interest regarding this manuscript.

## AUTHOR CONTRIBUTIONS

Project conception and organization: RZ, JLP, and BRZ. Acquisition of data and statistical analysis: RZ, CYJ, YC, YR, TG, ZHL, and JXD. First draft writing: RZ and JLP. Manuscript review and critique: RZ, CYJ, JLP, and BRZ. Approval of article and agreement for submission: RZ, JLP, and BRZ. All the co‐authors listed above gave their final approval of this manuscript version.

## Supporting information

Table S1‐S2
