# Supplementary material for: Analysis of rare variants of autosomal‐dominant genes in a Chinese population with sporadic Parkinson’s disease
Source: Mol Genet Genomic Med. 2020 Aug 14;8(10):e1449. doi: 10.1002/mgg3.1449 (PMC7549569; doi:10.1002/mgg3.1449)
Supplement: Supplementary file 1 — Table S1‐S2 [file MGG3-8-e1449-s001.docx]

**Appendix**

| **Supplementary table 1. Twelve AD-PD genes on the customized panel assessed for analytical validity.** | | | | |
| --- | --- | --- | --- | --- |
| Gene | Num_target_regions | Len of region | Target Fraction Region covered >1x | Target Average depth |
| SNCA | 6 | 6531 | 100.00% | 877 |
| LRRK2 | 51 | 11224 | 100.00% | 679 |
| GIGYF2 | 35 | 13139 | 100.00% | 922 |
| VPS35 | 17 | 6983 | 100.00% | 888 |
| EIF4G1 | 32 | 10623 | 100.00% | 912 |
| DNAJC13 | 57 | 9611 | 100.00% | 711 |
| CHCHD2 | 4 | 2966 | 100.00% | 948 |
| HTRA2 | 8 | 4620 | 100.00% | 812 |
| NR4A2 | 8 | 5531 | 100.00% | 980 |
| RIC3 | 9 | 9327 | 100.00% | 630 |
| TMEM230 | 7 | 4818 | 100.00% | 702 |
| UCHL1 | 9 | 3124 | 100.00% | 629 |

**Supplementary table 2. Single-variant analysis of all candidate variants**

| gene | chromosome position# | Amino Acid Change | Cases | | Controls | | P for HWE test (cases/Controls) | P | OR | 95%CI |
| --- | --- | --- | --- | --- | --- | --- | --- | --- | --- | --- |
|  |  |  | number | MAF | number | MAF |  |  |  |  |
| CHCHD2 | 56172037 | T61I | 1 | 5.24E-03 | 0 | 0.00E+00 | 0.97/- | - | - | - |
|  | 56174102 | P2L | 1 | 5.24E-03 | 1 | 5.00E-03 | 0.97/0.97 | 0.97 | 1.05 | 0.07-16.87 |
| DNAJC13 | 132181345 | L583S | 1 | 5.24E-03 | 0 | 0.00E+00 | 0.97/- | - | - | - |
|  | 132222104 | R1588H | 2 | 1.05E-02 | 0 | 0.00E+00 | 0.94/- | - | - | - |
| EIF4G1 | 184044759 | R1139H | 1 | 5.24E-03 | 2 | 1.00E-02 | 0.97/0.94 | 0.59 | 0.52 | 0.05-5.97 |
| GIGYF2 | 233684598 | E811A | 1 | 5.24E-03 | 0 | 0.00E+00 | 0.97/- | - | - | - |
|  | 233712060 | P1155T | 1 | 5.24E-03 | 0 | 0.00E+00 | 0.97/- | - | - | - |
| HTRA2 | 74757881 | T215M | 1 | 5.24E-03 | 0 | 0.00E+00 | 0.97/- | - | - | - |
| LRRK2 | 40646786 | A419V | 8 | 4.19E-02 | 0 | 0.00E+00 | 0.77/- | - | - | - |
|  | 40704252 | P1446L | 1 | 5.24E-03 | 1 | 5.00E-03 | 0.97/0.97 | 0.97 | 1.05 | 0.07-16.87 |
|  | 40704237 | R1441H | 1 | 5.24E-03 | 0 | 0.00E+00 | 0.97/- | - | - | - |
|  | 40702326 | I1339M | 2 | 1.05E-02 | 0 | 0.00E+00 | 0.94/- | - | - | - |
|  | 40713845 | R1628P | 10 | 5.24E-02 | 1 | 5.00E-03 | 0.71/0.97 | **0.005*** | 10.99 | 1.39-86.74 |
|  | 40692148 | R1067Q | 1 | 5.24E-03 | 0 | 0.00E+00 | 0.97/- | - | - | - |
| NR4A2 | 157182438 | V539M | 2 | 1.05E-02 | 0 | 0.00E+00 | 0.94/- | - | - | - |
|  | 157182309 | V582M | 1 | 5.24E-03 | 1 | 5.00E-03 | 0.97/0.97 | 0.97 | 1.05 | 0.07-16.87 |
| SNCA | 90756775 | V15D | 1 | 5.24E-03 | 1 | 5.00E-03 | 0.97/0.97 | 0.97 | 1.05 | 0.07-16.87 |
|  | 90650354 | M127I | 1 | 5.24E-03 | 0 | 0.00E+00 | 0.97/- | - | - | - |
| Total | - | - | 36 | 1.88E-01 | 7 | 3.50E-02 | - | **0.000*** | 6.40 | 2.77-14.79 |
| MAF, minor allele frequency; HWE, Hardy-Weinberg equilibrium; *, p<0.05. | | | | | | | | | | |
| #, Position on Genome Reference Consortium human genome build 37 (GRCh37). | | | | | | | | | | |
